# High-Level Constitutive Secretory Expression and Low-Temperature Adaptation Engineering of the Acid Protease RMAP9 from the Mangrove-Derived Yeast *Rhodotorula mucilaginosa*

**DOI:** 10.3390/biotech15040087

**Published:** 2026-10-06

**Authors:** Yibo Zhang, Jiasheng Cao, Mingming Luo, Zhijie Huang, Yu Tang, Zhizheng Zhao, Jian Lu

**Affiliations:** 1 College of Life Science and Technology, Guangxi University, Nanning 530004, China; zhangyibo1231@163.com (Y.Z.); aa1338213@163.com (J.C.); doutuy@163.com (Z.H.); yu.tang_0302@outlook.com (Y.T.); z2314701525@126.com (Z.Z.); 2 Institute of Biological Science and Technology, Guangxi Academy of Sciences, Nanning 530007, China; ro1347359103@gmail.com

**Keywords:** acid protease, *Komagataella phaffii*, secretion-signal engineering, constitutive expression, protein engineering

## Abstract

Acid proteases can improve nitrogen availability during ethanol fermentation by hydrolysing feedstock proteins, but their catalytic performance at relatively low temperatures may be limited. In this study, an acid protease-producing *Rhodotorula mucilaginosa* strain, TSM-6, was isolated from mangrove sediment, and an acid protease gene, *rmap9*, was identified by whole-genome sequencing and candidate-gene screening. RMAP9 was constitutively expressed in *Komagataella phaffii* GS115, and secretion was enhanced through EXG1-derived secretion-signal engineering, tandem assembly of three expression cassettes, and fermentation optimisation. Molecular dynamics simulations and Rosetta-based ΔΔG scoring were further used to identify candidate residues for temperature-adaptation engineering, followed by the site-saturation mutagenesis of Ser125. The optimised recombinant strain reached a maximum extracellular acid protease activity of 312.07 ± 18.37 U/mL, representing a 223% increase over the parental constitutive strain. The S125K variant showed a shift in optimum temperature from 50 to 40 °C and approximately 50% higher relative activity at 40 °C than wild-type RMAP9. Moreover, the low-temperature-favoured variants retained more than 80% residual activity after 72 h at 30 °C. These variants combined improved catalytic performance at relatively low temperatures with long-term stability at 30 °C, thereby providing enzyme properties compatible with temperature-related operational requirements for industrial ethanol fermentation.

## 1. Introduction

Acid proteases (EC 3.4.23) belong to the aspartic protease family and contain two highly conserved catalytic aspartate residues in their active sites, which cooperatively mediate acid–base catalysis to cleave peptide bonds in protein substrates [1]. Microbial acid proteases are attractive for industrial production because of their relatively short production cycles, the availability of low-cost fermentation substrates and their suitability for large-scale production [2]. These enzymes are widely used in food processing, animal feed production, pharmaceutical-related applications and the brewing industry [3]. In ethanol fermentation, urea is conventionally supplemented as a readily assimilable nitrogen source; however, urea can contribute to the formation of carcinogenic ethyl carbamate in the presence of ethanol, raising food-safety concerns [4]. Acid proteases can hydrolyse macromolecular proteins in fermentation feedstocks into small peptides and free amino acids that are readily assimilated by yeast as organic nitrogen sources. This can improve fermentation performance and ethanol production while reducing reliance on exogenous urea supplementation, making acid proteases a promising alternative strategy for nitrogen management in ethanol fermentation [5].

Commercial microbial acid proteases are predominantly associated with filamentous fungi, including Aspergillus, Rhizopus and other aspartic-protease-producing fungi [6]. However, conventional production with wild-type fungal strains may still be constrained by fermentation efficiency, strain productivity and downstream processing, creating a need for alternative production hosts and expression strategies. As unicellular eukaryotic microorganisms, yeasts combine relatively rapid growth and ease of cultivation and genetic manipulation with the protein-folding and post-translational modification machinery of eukaryotic cells, making them attractive hosts for the heterologous production of eukaryotic proteins [7]. Extracellular enzymes from marine-derived yeasts, including aspartic proteases, have been investigated, and several corresponding genes have been cloned and characterised [8]. For *R. mucilaginosa* specifically, the Antarctic marine strain L7 has previously been used for native extracellular acid-protease production, purification and biochemical characterisation, followed by culture optimisation that increased proteolytic activity by approximately 45% [9,10]. These studies established the biotechnological potential of *R. mucilaginosa* proteases but focused primarily on native-host production and enzyme characterisation. In contrast, the novelty of the present study lies in integrating genome-guided candidate screening with methanol-free constitutive production, secretion-signal and tandem-cassette engineering, fermentation optimisation, and temperature-adaptation engineering of the gene-defined enzyme RMAP9. This integrated strategy is particularly relevant to mangrove-derived yeasts, whose enzyme resources remain incompletely explored [11].

*Komagataella phaffii* (formerly *Pichia pastoris*) is one of the most widely used eukaryotic hosts for heterologous protein production, owing to its high-density cultivation capacity, efficient secretion of heterologous proteins, low levels of endogenous extracellular proteins and suitability for cost-effective production [12]. The commonly used AOX1 promoter enables strong inducible expression but requires methanol as an inducer, increasing the complexity of substrate handling, feeding control and process operation. By contrast, the constitutive GAP promoter does not require an exogenous inducer and can therefore simplify fermentation operation, although its expression performance depends on the host background, target protein and cultivation conditions [13]. In addition to promoter selection, the final extracellular production of a heterologous protein in *K. phaffii* is influenced by protein folding and intracellular trafficking [14], secretion-signal efficiency [15], expression-cassette dosage [16], and cultivation conditions [17]. Accordingly, integrating secretion-signal engineering, increased expression-cassette dosage and fermentation optimisation represents a practical strategy for enhancing constitutive extracellular production of RMAP9.

The optimum reaction temperatures of several characterised fungal aspartic proteases fall within the range of approximately 50–60 °C [18], whereas conventional *Saccharomyces cerevisiae*-based ethanol fermentation is generally operated at substantially lower temperatures, typically around 30–37 °C [19]. Consequently, acid proteases with high optimum temperatures may exhibit reduced catalytic performance under typical ethanol-fermentation conditions. Engineering acid proteases to shift their optimum reaction temperatures towards lower values while enhancing catalytic activity at relatively low temperatures is therefore important for improving their compatibility with industrial fermentation processes and broadening their applications [20]. Molecular engineering of microbial aspartic proteases has addressed catalytic efficiency, stability and production performance; however, targeted engineering specifically aimed at improving catalytic performance at relatively low temperatures has been less frequently reported [21]. Semi-rational design approaches integrating protein structure modelling and molecular dynamics simulations can facilitate the identification of amino acid residues associated with enzyme function and temperature adaptation [22]. Focused or site-saturation mutagenesis can subsequently be used to explore beneficial substitutions at selected positions [23]. The integration of computational analysis with experimental screening provides an effective strategy for tailoring enzyme properties [24].

In this study, an acid protease-producing yeast was isolated from mangrove sediment in the Beilun Estuary, Guangxi, China, and the acid protease gene *rmap9* was subsequently cloned. RMAP9 was then used as the target enzyme for systematic optimisation of heterologous production and molecular engineering. Endogenous secretion signals from *K. phaffii* were introduced to engineer the α-factor-based secretion system, and the most effective secretion-signal combination was identified. On this basis, constructs containing tandem expression cassettes were generated to further enhance extracellular production of RMAP9. The fermentation medium and cultivation conditions were subsequently optimised through single-factor and orthogonal experiments to improve extracellular acid protease production. In parallel, protein structure modelling and molecular dynamics simulations were employed to identify candidate residues associated with temperature adaptation, followed by site-saturation mutagenesis for experimental screening of beneficial variants. Through these combined strategies, a high-level constitutive production system and RMAP9 variants with improved catalytic performance at relatively low temperatures were obtained, providing a basis for the industrial application of RMAP9, particularly in ethanol fermentation.

## 2. Materials and Methods

### 2.1. Materials

Mangrove sediment samples were collected from the land–sea interface within the Beilun Estuary mangrove area in Fangchenggang, Guangxi, China (108.10° E, 21.55° N). *Escherichia coli* DH5α was used for gene cloning and plasmid propagation, whereas *Komagataella phaffii* GS115 was used as the host for heterologous expression. The pMD19-T vector (Amp^r^) was used for gene cloning and sequence verification, while pPIC9K (Amp^r^) and pGAPZα-A (Zeocin^r^) were used for heterologous expression in *K. phaffii*. T4 DNA ligase, PrimeSTAR HS DNA polymerase, and the restriction endonucleases *Eco*RI, *Not*I, *Bln*I, *Bam*HI, *Bgl*II, *Stu*I, *Dpn*I, *Avr*II and *Sca*I were purchased from Takara Bio (Beijing, China). The yeast genomic DNA extraction kit, plasmid DNA miniprep kit, DNA purification kit and seamless cloning kit were purchased from Sangon Biotech (Shanghai, China). The total RNA extraction kit and first-strand cDNA synthesis kit were obtained from Nanjing Yifeixue Biotechnology Co., Ltd. (Nanjing, China). Folin–Ciocalteu reagent, Zeocin, casein and bovine serum albumin were purchased from Solarbio (Beijing, China). The 2× RealStar Fast SYBR qPCR Mix was obtained from GenStar(Beijing, China).

### 2.2. Methods

#### 2.2.1. Isolation of Acid Protease-Producing Strains

A 10.0 g portion of the collected mangrove sediment sample was suspended in 90 mL of sterile 0.9% (*w*/*v*) NaCl solution containing glass beads and agitated at 30 °C and 150 r/min for 1 h. The suspension was then transferred to yeast extract–peptone–dextrose (YPD) broth supplemented with 100 μg/mL chloramphenicol and incubated at 30 °C and 150 r/min for 48 h. The resulting culture was serially diluted and spread onto acidified skimmed-milk agar plates, which were incubated in an inverted position at 30 °C for 48 h. Protease-positive colonies were repeatedly streaked onto fresh plates until pure cultures were obtained. The purified isolates were preserved as glycerol stocks at −80 °C. Each isolate was subsequently inoculated into fermentation medium at an inoculum size of 2% (*v*/*v*) and cultivated at 30 °C and 150 r/min for 48 h. After centrifugation at 8000 r/min, the culture supernatant was collected, and acid protease activity was determined using the Folin–Ciocalteu method. The isolate exhibiting the highest extracellular acid protease activity, designated TSM-6, was selected for subsequent experiments.

#### 2.2.2. Identification of the Acid Protease-Producing Strain

The TSM-6 isolate obtained after secondary screening was cultured on YPD agar plates to examine colony morphology and pigmentation, and its cellular morphology was subsequently observed by light microscopy. Carbon assimilation was evaluated using glucose, sucrose, cellobiose, maize xylan, maltose, soluble starch, raffinose, α-lactose and galactose according to standard physiological and biochemical identification procedures. Genomic DNA was extracted from strain TSM-6 using a yeast genomic DNA extraction kit. The 18S rRNA gene, the internal transcribed spacer (ITS) region, and the D1/D2 domain of the 26S rRNA gene were amplified using universal primers. The PCR products were ligated into the pMD19-T vector, and positive clones were subjected to sequencing by Sangon Biotech (Shanghai, China). The resulting sequences were analysed for sequence similarity using NCBI BLAST. Relevant reference sequences were retrieved and aligned using Clustal X (version 2.1). A phylogenetic tree based on the 26S rRNA gene was constructed using the neighbour-joining method in MEGA 11, with branch support assessed using 1000 bootstrap replicates.

#### 2.2.3. Whole-Genome Sequencing and Bioinformatic Analysis

Genomic DNA was extracted from strain TSM-6 and subjected to de novo whole-genome sequencing on an Illumina platform by Sangon Biotech (Shanghai, China), generating a draft genome assembly of strain TSM-6. Functional annotation was performed by searching the predicted sequences against the Gene Ontology (GO), KOG, COG, KEGG, Conserved Domain Database (CDD), NR, NT, Pfam, Swiss-Prot and TrEMBL databases. The physicochemical properties of the deduced amino acid sequences were analysed using ExPASy ProtParam. Signal peptides were predicted using SignalP 4.1, and putative functional sites and conserved domains were analysed using SMART and PROSITE.

#### 2.2.4. Cloning of the Acid Protease Gene and Construction of Expression Vectors

##### Full-Length cDNA Cloning

Total RNA was extracted from strain TSM-6 using a total RNA extraction kit and reverse-transcribed to obtain first-strand cDNA. PCR amplification was performed under the following conditions: initial denaturation at 95 °C for 3 min, 30 cycles of denaturation at 94 °C for 30 s, annealing at 65 °C for 30 s and extension at 72 °C for 2 min, followed by a final extension at 72 °C for 10 min. The PCR products were analysed by electrophoresis on a 1% agarose gel, and the target fragments were excised and purified from the gel. The purified fragments were ligated into the pMD19-T vector and transformed into *E. coli* DH5α. Positive clones were verified by colony PCR and sequencing. The five successfully cloned genes (*rmap3*, *rmap5*, *rmap7*, *rmap9* and *rmap10*) were subsequently subjected to heterologous expression screening in *K. phaffii* GS115. Recombinant strains were verified by genomic PCR, and candidate-protein expression was examined by SDS-PAGE of culture supernatants and, where required, cell lysates.

##### Construction of the Inducible Expression Vector pPIC9K-*rmap9*

Based on the sequence analysis, primers were designed to amplify the *rmap9* coding sequence lacking its native signal peptide, using pMD19-T-*rmap9* as the template. The forward primer F-EcoRI was 5′-CCGGAATTCGCTCCCGTCGAGACCCT-3′, and the reverse primer R-NotI was 5′-CTAGTTTAGCGGCCGCCTAGTGGTGGTGGTGGTGGTGAGCG-3′. The purified PCR product and pPIC9K vector were digested with EcoRI and NotI and subsequently ligated using T4 DNA ligase. The ligation products were transformed into *E. coli* DH5α, and the resulting recombinant clones were verified by sequencing at Sangon Biotech (Shanghai, China). The verified recombinant plasmid was designated pPIC9K-*rmap9*. The plasmid was linearised with BglII, purified and subsequently used for electroporation into *K. phaffii* GS115.

##### Construction of the Constitutive Expression Vector pGAPZα-A-*rmap9*

The pPIC9K-*rmap9* plasmid and pGAPZα-A vector were digested with EcoRI and NotI, and the *rmap9* insert and linearised pGAPZα-A vector were recovered and purified. The two fragments were ligated using T4 DNA ligase and transformed into *E. coli* DH5α. Transformants were spread onto low-salt LB agar plates (pH 7.5) containing 25 μg/mL Zeocin and incubated at 37 °C for 12–16 h. Positive recombinant clones were selected and verified by sequencing at Sangon Biotech (Shanghai, China). The resulting recombinant plasmid was designated pGAPZα-A-*rmap9*. The plasmid was linearised with AvrII, purified and subsequently used for electroporation into *K. phaffii* GS115. For methanol-inducible production, recombinant cells were cultivated in BMMY at 28 °C and 250 rpm, with methanol added every 24 h to a final concentration of 1.0% (*v*/*v*). For constitutive production, recombinant cells were cultivated in YPD at 30 °C and 250 rpm.

#### 2.2.5. Secretion-Signal Engineering and Construction of Recombinant Strains

Endogenous pre-regions derived from DSE4, EXG1, FLO10 and PHO5 were individually used to replace the native pre-region of the α-factor prepro-leader in pGAPZα-A-*rmap9* by seamless cloning, while retaining the α-factor pro-region. The resulting recombinant vectors were linearised with BlnI and electroporated into *K. phaffii* GS115. Positive transformants were selected on the basis of Zeocin resistance and verified by PCR.

To minimise the effect of gene copy number on secretion-signal comparison, three positive transformants from each construct were randomly selected for copy-number determination by qPCR. Standard curves for *gap* and *rmap9* were generated using pMD19-T-gap and pMD19-T-*rmap9* plasmids diluted to 10^4^–10^8^ copies/μL. Genomic qPCR was performed using RTgap-F/RTgap-R and RT*rmap9*-F/RT*rmap9*-R. Copy numbers of *gap* and *rmap9* were calculated from the corresponding standard curves, and the *rmap9/gap* ratio was used to identify single-copy transformants. For secretion-signal comparison, seed cultures of the selected single-copy transformants were adjusted to the same OD_600_ before inoculation at 1% (*v*/*v*). Culture supernatants from the selected secretion-signal strains were collected at 72 h, and equal volumes of the supernatants were subjected to SDS-PAGE for the qualitative comparison of extracellular RMAP9 accumulation.

#### 2.2.6. Construction of Tandem Expression-Cassette Vectors

The expression cassette containing the optimised chimeric secretion signal, designated GAP-*rmap9*-EXG1, was excised from the recombinant vector using BamHI and BglII, which generate compatible cohesive ends. The cassette was sequentially ligated into the pPIC9K vector to construct recombinant plasmids containing one, two or three tandem expression cassettes. The resulting one-, two- and three-cassette plasmids were verified by restriction digestion, linearised with StuI, and electroporated into *K. phaffii* GS115. The cassette numbers therefore refer to the verified plasmid architecture before transformation; the genomic copy numbers and integration loci of the resulting transformants were not independently determined. Recombinant transformants were subsequently selected on the basis of antibiotic resistance.

#### 2.2.7. Optimisation of Fermentation Medium and Cultivation Conditions

The recombinant strain GS115/pPIC9K-(GAP-*rmap9*-EXG1)_3_, generated using the three-cassette construct, was used for shake-flask fermentation optimisation. Single-factor experiments evaluated carbon source, maltose concentration (2–12%, *w*/*v*), nitrogen-source combinations and concentrations, and mineral salts (0.1%, *w*/*v*). Based on these results, glucose (6%, 8% and 10%, *w*/*v*), beef extract (4%, 6% and 8%, *w*/*v*) and yeast extract (2%, 3% and 4%, *w*/*v*) were further optimised using an L_9_(3^4^) orthogonal array. The optimised medium was subsequently used to evaluate cultivation temperature (28–37 °C), initial pH (2.0–7.0) and inoculum size (1–5%, *v*/*v*) by single-factor experiments. Each treatment was performed in triplicate, with extracellular acid protease activity in the fermentation supernatant used as the response variable.

#### 2.2.8. Protein Structure Modelling, Molecular Dynamics Simulations and Mutagenesis of RMAP9

The three-dimensional structure of RMAP9 was predicted using trRosetta [25]. The predicted model was subsequently used as the starting structure for 100 ns molecular dynamics (MD) simulations at 30 and 35 °C using GROMACS 2019.6 [26] with the CHARMM36m force field [27]. RMSD trajectories were analysed to evaluate the overall conformational behaviour of RMAP9 during the simulations. Residue-wise RMSF values were calculated to assess local structural flexibility, and temperature-dependent changes in residue flexibility were further expressed as ΔRMSF, defined as the RMSF at 35 °C minus that at 30 °C. Residue-wise ΔRMSF analysis was used to screen candidate positions for subsequent evaluation. Candidate substitutions were assessed using Rosetta3 [28] with a ΔΔG-based stability-evaluation protocol [29]. A75G, Y85G and S125A were generated by inverse PCR for preliminary biochemical evaluation. Site-saturation mutagenesis was subsequently performed at Ser125.

#### 2.2.9. Enzyme Activity Assay and Biochemical Characterisation

Wild-type RMAP9 and its variants, each carrying a C-terminal 6×His tag, were purified using the same procedure. Fermentation broths were centrifuged at 8000 rpm for 10 min to remove cells, and the resulting supernatants were concentrated using 10-kDa molecular-weight-cutoff centrifugal ultrafiltration devices. The concentrated samples were subjected to buffer exchange into binding buffer containing 50 mM NaH_2_PO_4_, 300 mM NaCl and 10 mM imidazole (pH 8.0) by repeated ultrafiltration. The samples were then incubated with Ni-NTA resin to allow binding of the His-tagged recombinant proteins. Unbound and nonspecifically bound proteins were removed using wash buffer containing 50 mM NaH_2_PO_4_, 300 mM NaCl and 20 mM imidazole (pH 8.0). The bound recombinant proteins were subsequently eluted with elution buffer containing 50 mM NaH_2_PO_4_, 300 mM NaCl and 250 mM imidazole (pH 8.0). The eluates were subjected to a second ultrafiltration-based buffer-exchange step before biochemical characterisation. The C-terminal 6×His tag was retained throughout the subsequent biochemical assays, and protein purity was assessed qualitatively by SDS-PAGE. Acid protease activity was determined using a modified Folin–Ciocalteu colorimetric method with casein as the substrate. Briefly, 200 μL of 1% (*w*/*v*) casein prepared in 0.05 M glycine–HCl buffer (pH 2.5) was pre-incubated for 5 min, followed by the addition of 20 μL of appropriately diluted enzyme sample. Unless otherwise specified, the reaction was performed at 50 °C and pH 2.5 for 20 min and terminated by adding 200 μL of 0.4 M trichloroacetic acid. After centrifugation at 8000 rpm for 5 min, 100 μL of the resulting supernatant was mixed with 500 μL of 0.4 M Na_2_CO_3_ and 100 μL of Folin–Ciocalteu reagent. Colour development was carried out at 40 °C for 20 min, and the absorbance was measured spectrophotometrically at 680 nm using a Tecan microplate reader. Enzyme activity was quantified against an L-tyrosine standard curve. One unit of enzyme activity (U) was defined as the amount of enzyme required to release 1 μg of tyrosine per minute from casein under the specified assay conditions.

The biochemical properties of the purified wild-type enzyme and its variants were subsequently characterised. The optimum reaction temperature was determined over a range of 30–70 °C, and the optimum pH was determined over a range of pH 1.5–3.5. For kinetic analysis, initial reaction rates were measured using casein concentrations of 0.25, 0.5, 0.75, 1.0, 1.5, 2.0, 2.5, 3.0, 5.0, 8.0 and 10.0 mg/mL under the optimum reaction conditions of each enzyme. The kinetic data were analysed by nonlinear regression using GraphPad Prism 8.0 and fitted to the Michaelis–Menten equation to obtain the apparent Michaelis constant (*K*_m_) and maximum reaction velocity (*V*_max_). The catalytic constant (*k*_cat_) was subsequently calculated from the fitted *V*_max_ and the molar amount of enzyme, and catalytic efficiency was expressed as *k*_cat_/*K*_m_.

Thermal stability at 30 °C was evaluated by incubating appropriately diluted purified enzyme preparations at 30 °C for different periods over 72 h. A corresponding enzyme aliquot maintained at 4 °C was used as the activity reference and defined as 100%. After incubation, residual protease activity was measured under the optimum reaction conditions of the corresponding enzyme using the casein–Folin–Ciocalteu assay described above. Residual activity was expressed as a percentage of the reference activity and was used as the functional readout of enzyme stability at 30 °C.

#### 2.2.10. Statistical Analysis

Unless otherwise stated, experiments were performed in triplicate and data are presented as the mean ± standard deviation. Statistical analyses were performed using GraphPad Prism 8.0. Where inferential comparisons were applicable, datasets involving a single experimental factor were analysed by one-way analysis of variance (ANOVA), whereas datasets involving two experimental factors were analysed by two-way ANOVA; Tukey’s multiple-comparisons test was used for post hoc comparisons. Differences were considered statistically significant at *p* < 0.05.

## 3. Results and Discussion

### 3.1. Screening and Identification of the Acid Protease-Producing Strain

An acid protease-producing strain was isolated from mangrove sediment collected from the Beilun Estuary in Fangchenggang, Guangxi, China, and was designated TSM-6. After 48 h of cultivation on YPD agar, strain TSM-6 formed pink, circular and convex colonies with smooth, moist surfaces (Figure S1A). Microscopic examination showed that the cells were oval in shape and reproduced by budding (Figure S1B). Carbon assimilation tests showed that strain TSM-6 could utilise glucose, sucrose, galactose, α-lactose and soluble starch, but not cellobiose, raffinose, maltose or maize xylan (Table S1).

Genomic DNA was extracted from strain TSM-6, and the 18S rRNA gene, internal transcribed spacer (ITS) region and D1/D2 domain of the 26S rRNA gene were amplified by PCR and sequenced (Figure S2). The resulting sequences were analysed using NCBI BLASTn, and a phylogenetic tree was subsequently constructed using MEGA 11 (Figure S3). Sequence similarity analysis of the 18S rRNA, ITS and 26S D1/D2 sequences, together with phylogenetic placement based on the 26S D1/D2 region, supported the identification of strain TSM-6 as *R. mucilaginosa*. Whole-genome sequencing of TSM-6 followed by functional annotation identified 10 putative acid protease genes (Table S2). Multiple sequence alignment of their deduced amino acid sequences using DNAMAN revealed that all contained the conserved Asp-Thr/Ser-Gly motif characteristic of aspartic proteases, although their overall sequence similarity was relatively low (Figure S4). The isolation of *R. mucilaginosa* TSM-6 expands the available resources for exploring acid proteases from environmentally specialised yeasts.

### 3.2. Heterologous Expression of Acid Proteases

**Figure 1:**
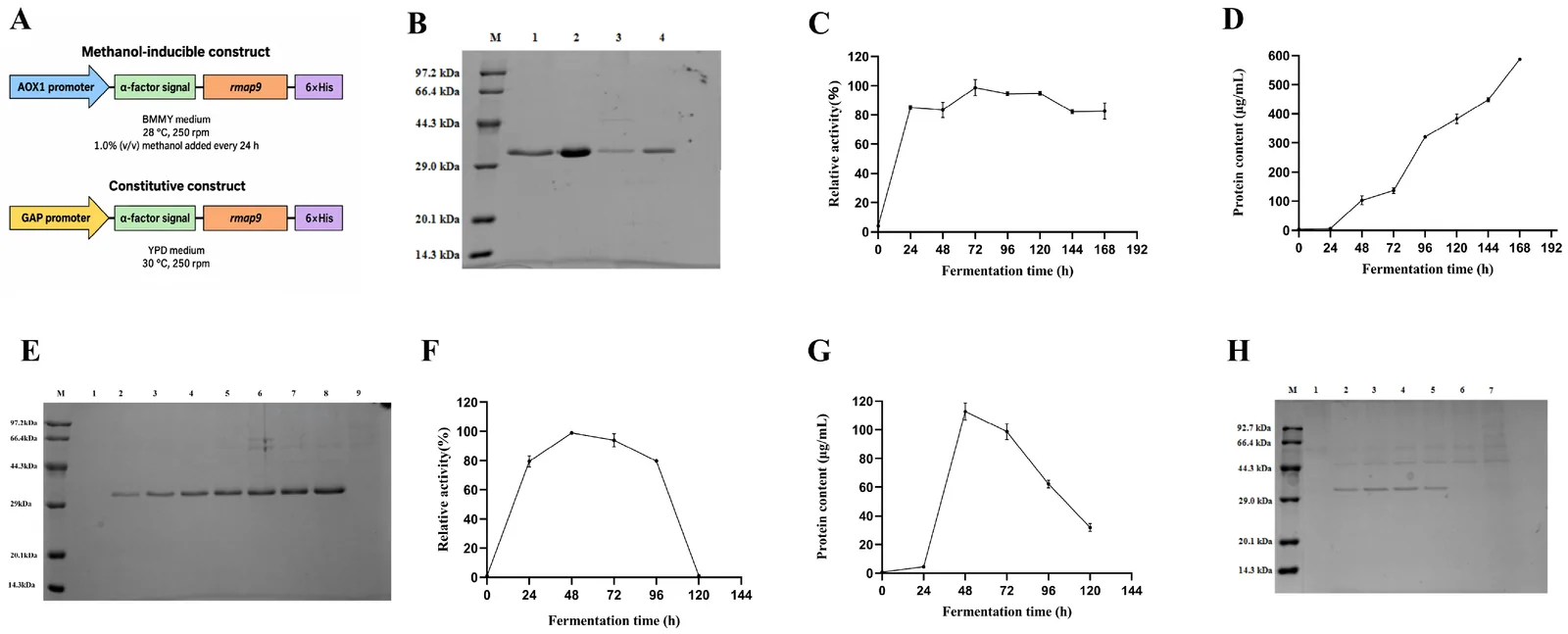
Expression constructs, purification, and time-dependent production profiles of RMAP9 in *K. phaffii*. (**A**) Schematic representation of the methanol-inducible and constitutive RMAP9 expression constructs and the corresponding major shake-flask cultivation conditions. (**B**) SDS-PAGE analysis of RMAP9 before and after Ni-NTA affinity purification. M, protein molecular-weight marker; lane 1, fermentation supernatant of methanol-inducible RMAP9; lane 2, purified methanol-inducible RMAP9; lane 3, fermentation supernatant of constitutively expressed RMAP9; lane 4, purified constitutively expressed RMAP9. (**C**) Relative extracellular acid protease activity of GS115/pPIC9K-*rmap9* during methanol-inducible cultivation. (**D**) Total extracellular protein concentration in the corresponding culture supernatant. (**E**) Time-course SDS-PAGE analysis of the fermentation supernatant of GS115/pPIC9K-*rmap9*. M, protein molecular-weight marker; lanes 1–8, culture supernatants collected at 0, 24, 48, 72, 96, 120, 144, and 168 h, respectively; lane 9, fermentation supernatant of the GS115/pPIC9K vector control. (**F**) Relative extracellular acid protease activity of GS115/pGAPZα-A-*rmap9* during constitutive cultivation. (**G**) Total extracellular protein concentration in the corresponding culture supernatant. (**H**) Time-course SDS-PAGE analysis of the fermentation supernatant of GS115/pGAPZα-A-*rmap9*. M, protein molecular-weight marker; lanes 1–6, culture supernatants collected at 0, 24, 48, 72, 96, and 120 h, respectively; lane 7, fermentation supernatant of the GS115/pGAPZα-A vector control.

Based on the whole-genome annotation of *R. mucilaginosa* TSM-6, cDNA cloning was attempted for the 10 putative acid protease genes, of which five (*rmap3*, *rmap5*, *rmap7*, *rmap9* and *rmap10*) were successfully obtained. All five recombinant strains were confirmed by genomic PCR and subjected to methanol-inducible expression screening. Only the *rmap9* strain showed detectable proteolytic activity. SDS-PAGE showed extracellular bands of the expected sizes for RMAP7 and RMAP9, whereas no obvious target bands were detected for RMAP3, RMAP5 or RMAP10 in either culture supernatants or cell lysates (Figure S5). Thus, RMAP7 was secreted but lacked detectable proteolytic activity, whereas no target proteins corresponding to RMAP3, RMAP5 or RMAP10 were detectable by SDS-PAGE under the conditions tested. RMAP9 was therefore selected for subsequent characterisation. The expression constructs and major shake-flask cultivation conditions for the methanol-inducible and constitutive systems are summarised (Figure 1A). Representative SDS-PAGE analysis following Ni-NTA affinity purification showed a predominant protein band at approximately 34 kDa for RMAP9 produced by both expression systems, consistent with the expected molecular mass of the mature recombinant enzyme (Figure 1B).

In the methanol-inducible system, extracellular acid protease activity increased following methanol induction and reached its maximum at 72 h (Figure 1C). Total extracellular protein in the culture supernatant showed a different temporal profile and continued to increase during cultivation (Figure 1D). Time-course SDS-PAGE analysis showed that no obvious RMAP9 band was present at 0 h, whereas an approximately 34-kDa band became detectable at 24 h and accumulated during subsequent cultivation (Figure 1E).

Subsequently, the constitutive expression vector pGAPZα-A-*rmap9*, in which *rmap9* was driven by the GAP promoter, was constructed and introduced into *K. phaffii* GS115. In the resulting constitutive strain, extracellular acid protease activity reached its maximum at 48 h (Figure 1F), whereas total extracellular protein also peaked at approximately 48 h and subsequently declined (Figure 1G). Time-course SDS-PAGE further visualised extracellular RMAP9 at individual cultivation time points from 0 to 120 h (Figure 1H). Importantly, total extracellular protein represents all proteins present in the culture supernatant rather than RMAP9 specifically; therefore, it should not be expected to correlate directly or linearly with RMAP9-specific protease activity.

The methanol-inducible AOX1 system requires additional inducer handling and feed control, thereby increasing fermentation complexity and process-management requirements. In contrast, constitutive or other methanol-free expression strategies can simplify fermentation operation, although their productivity remains dependent on promoter architecture, the target protein and cultivation conditions [30]. Accordingly, the extracellular production of RMAP9 obtained with the initial GAP-based construct still had considerable scope for improvement, suggesting that secretion-signal engineering and increased expression-cassette dosage could provide complementary routes for further enhancement.

### 3.3. Effects of Secretion-Signal Engineering on the Extracellular Production of RMAP9

**Figure 2:**
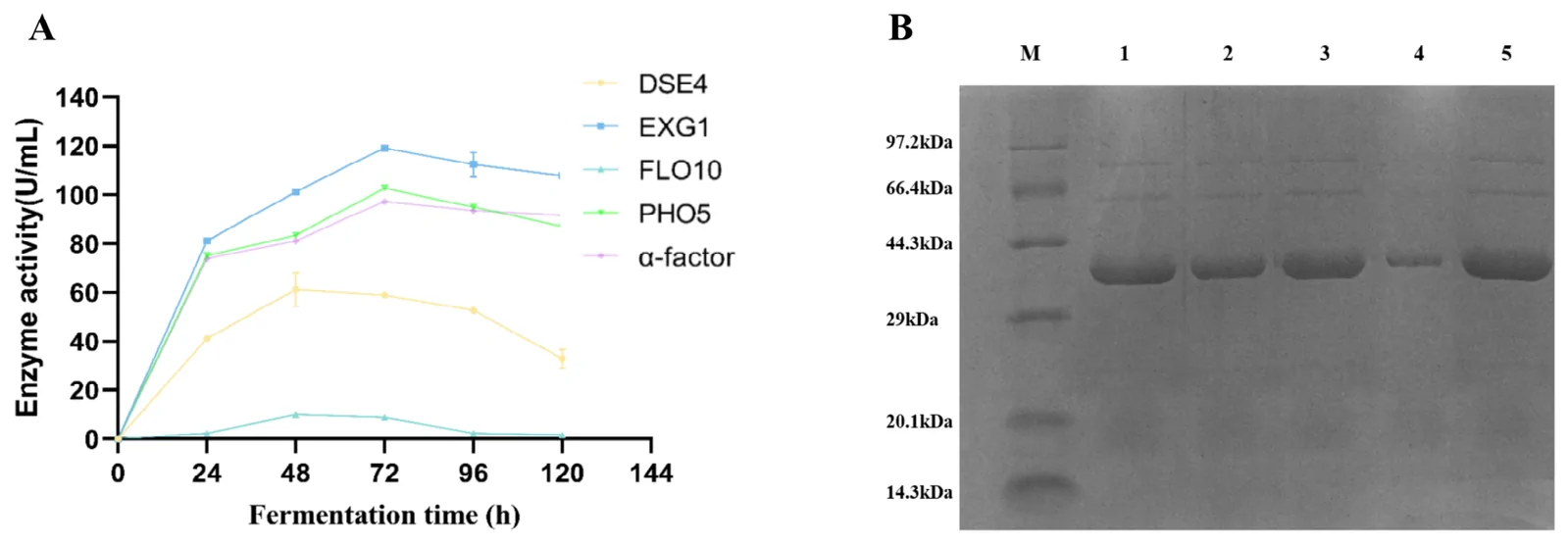
Effects of different secretion signals on extracellular RMAP9 production. (**A**) Extracellular acid protease activities of recombinant strains carrying different secretion signals. (**B**) SDS-PAGE analysis of equal volumes of the corresponding culture supernatants collected at 72 h. M, protein molecular-weight marker; lanes 1–5, α-factor, DSE4, PHO5, FLO10 and EXG1, respectively.

To further enhance the extracellular production of RMAP9 in the constitutive expression system, four endogenous pre-regions derived from DSE4, EXG1, FLO10 and PHO5 were individually used to replace the α-factor pre-region while retaining its pro-region. The strain containing the original α-factor secretion leader exhibited an extracellular acid protease activity of 96.52 ± 2.53 U/mL at 72 h. Among the engineered constructs, the EXG1-derived secretion signal increased extracellular enzyme activity by approximately 24%, whereas the PHO5-derived construct produced an activity comparable to that of the original α-factor-based construct, and the DSE4-derived construct resulted in an approximately 36% decrease. The FLO10-derived construct also exhibited markedly lower extracellular acid protease activity than the original α-factor-based construct throughout cultivation (Figure 2A). Consistent with these activity profiles, SDS-PAGE analysis of the culture supernatants showed that the approximately 34-kDa RMAP9 band was strongest for the EXG1-derived construct, comparable between the α-factor- and PHO5-derived constructs, weaker for DSE4, and faintest for FLO10 (Figure 2B). These results indicate that the signal-dependent differences in extracellular activity were associated with differences in extracellular RMAP9 accumulation.

Secretion-signal performance in *K. phaffii* is strongly dependent on the specific signal sequence–target protein combination, and systematic screening studies have shown that alternative or endogenous secretion signals can produce markedly different secretion efficiencies for different recombinant proteins [31,32]. In the present constructs, the GAP promoter, α-factor pro-region and *rmap9* coding sequence were retained, whereas only the pre-region was replaced. Moreover, single-copy transformants were used and the seed cultures were normalised to the same OD_600_ before inoculation. The higher extracellular RMAP9 accumulation observed with the EXG1-derived construct may therefore reflect a more favourable compatibility between its pre-region and RMAP9 during the early stages of the secretory pathway. The pre-region primarily mediates targeting and translocation into the endoplasmic reticulum, and alterations in secretion signals have been shown to influence translocation efficiency, unfolded-protein-response activation and intracellular mis-sorting or degradation [33]. Consistent with this interpretation, recent secretion-pathway engineering in *P. pastoris* demonstrated that improving ER translocation and unfolded-protein-response capacity can substantially enhance extracellular recombinant protein production [34]. Thus, the lower activities observed with the DSE4- and FLO10-derived constructs may reflect less efficient ER targeting, translocation and/or subsequent processing. Although the relative contributions of ER stress, protein folding and growth during cultivation were not directly resolved in the present study, these mechanisms provide a biologically plausible framework for the signal-dependent differences observed here.

### 3.4. Effect of Tandem Expression-Cassette Constructs on Extracellular Acid Protease Production

**Figure 3:**
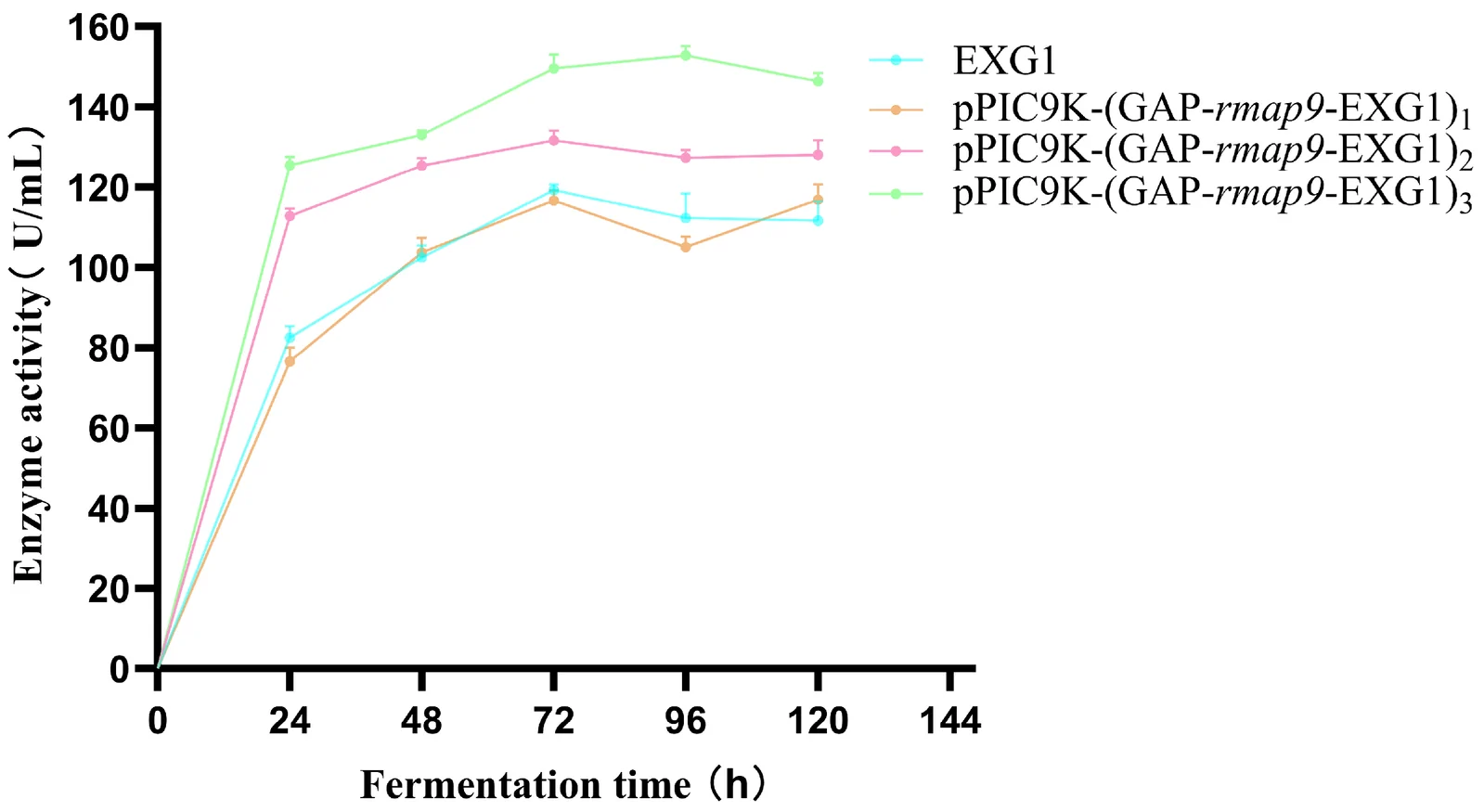
Effect of tandem expression-cassette constructs on extracellular acid protease production.

Based on the superior performance of the EXG1-derived secretion construct, recombinant strains were further generated using constructs containing one, two or three tandem GAP-*rmap9*-EXG1 expression cassettes. Extracellular acid protease activity increased progressively among strains generated from the one-, two- and three-cassette constructs. The strain generated from the single-cassette construct exhibited an activity of 116.23 ± 5.14 U/mL, whereas that generated from the two-cassette construct reached 133.66 ± 4.24 U/mL, representing an increase of approximately 15% over the single-cassette construct-derived strain. The strain generated from the three-cassette construct showed the highest extracellular activity of 152.85 ± 2.06 U/mL, corresponding to an increase of approximately 31% relative to the single-cassette construct-derived strain (Figure 3).

Increasing gene dosage is a common strategy for enhancing recombinant protein production in *K. phaffii* [35], whereas excessive expression can induce unfolded-protein and secretory stress and thereby limit further productivity gains [36]. Recombinant productivity can also be influenced by the genomic integration event [37], and recent studies have shown that high heterologous expression loads may reduce biomass accumulation and growth rate in *K. phaffii* [38]. In the present study, extracellular activity increased from 116.23 ± 5.14 to 152.85 ± 2.06 U/mL among strains generated from the one- to three-cassette constructs. However, because genomic copy number, integration locus and growth during cultivation were not independently quantified, these differences are interpreted as effects associated with the tandem-cassette constructs rather than being attributed solely to verified genomic gene dosage.

### 3.5. Optimisation of Fermentation Medium and Cultivation Conditions

**Figure 4:**
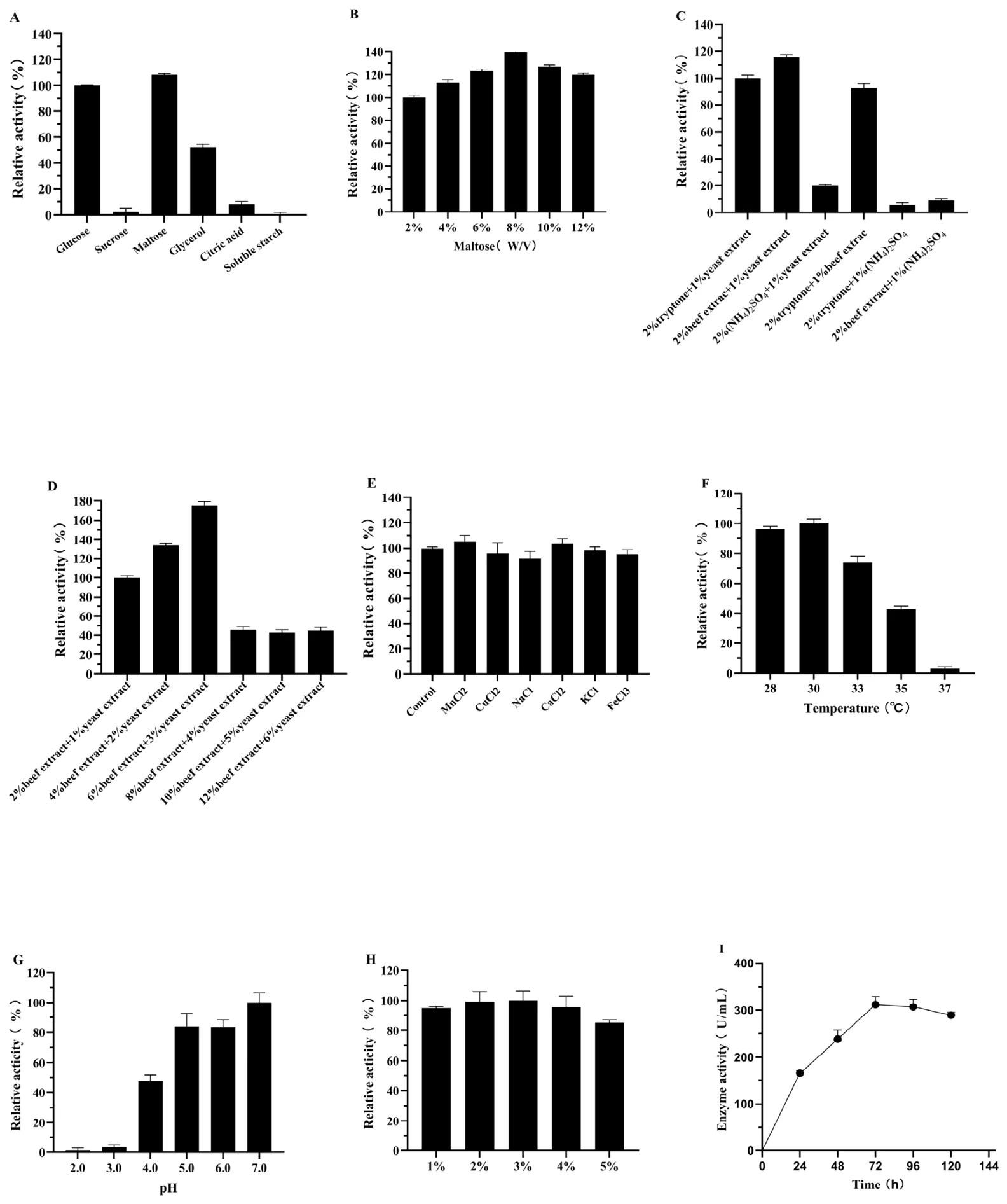
Optimisation of medium composition and shake-flask cultivation conditions for extracellular RMAP9 production: (**A**) effect of carbon source; (**B**) effect of maltose concentration; (**C**) effect of nitrogen-source combinations; (**D**) effect of beef-extract and yeast-extract concentrations; (**E**) effect of mineral salts; (**F**) effect of cultivation temperature; (**G**) effect of initial pH; (**H**) effect of inoculum size; (**I**) extracellular acid protease activity during cultivation under the optimised conditions.

The recombinant strain GS115/pPIC9K-(GAP-*rmap9*-EXG1)_3_, generated using the three-cassette construct, was used for fermentation optimisation. Single-factor experiments were first conducted to investigate the effects of carbon source and concentration, nitrogen source and concentration, and mineral salts. Maltose supported the highest extracellular acid protease activity among the carbon sources tested (Figure 4A), with an optimum concentration of 8% (*w*/*v*) (Figure 4B). However, considering the higher cost of maltose and its limited advantage in enzyme production, glucose was selected as the carbon source for subsequent optimisation because of its lower cost and greater suitability for large-scale fermentation.

Among the nitrogen-source combinations tested, a mixture of 2% (*w*/*v*) beef extract and 1% (*w*/*v*) yeast extract resulted in the highest extracellular enzyme activity (Figure 4C). Further optimisation showed that the optimal concentrations of beef extract and yeast extract were 6% and 3% (*w*/*v*), respectively (Figure 4D). Supplementation with mineral salts, including KCl and CaCl_2_, did not appreciably improve enzyme production (Figure 4E). Based on these results, glucose (A), beef extract (B), and yeast extract (C) were selected for further optimisation using an L_9_(3^4^) orthogonal array (Table 1). Range analysis indicated that the relative effects of the three factors on extracellular acid protease production followed the order beef extract > yeast extract > glucose. The highest activity in the orthogonal array was obtained with A3B1C3, corresponding to 100 g/L glucose, 40 g/L beef extract and 40 g/L yeast extract; this combination was therefore selected for subsequent cultivation optimisation.

Fermentation temperature, initial pH and inoculum size were subsequently optimised by single-factor experiments. The optimal shake-flask cultivation conditions were 30 °C, an initial pH of 7.0 and an inoculum size of 2% (*v*/*v*) (Figure 4F–H). Under the optimised medium and cultivation conditions, the recombinant strain achieved a maximum extracellular acid protease activity of 312.07 ± 18.37 U/mL after 72 h of shake-flask fermentation, representing an approximately 104% increase compared with the activity before fermentation optimisation (152.85 ± 2.06 U/mL) (Figure 4I).

Medium composition and cultivation conditions are important determinants of heterologous protein production in *K. phaffii* [39]. Although maltose supported slightly higher extracellular enzyme activity in the initial screening, its activity advantage over glucose was limited. Considering enzyme production together with process economics and practical operability, glucose was therefore selected for subsequent medium optimisation. Beef extract and yeast extract provided an effective combined nitrogen source in the present experiments, and orthogonal optimisation further defined an appropriate combination of the three principal medium components for extracellular RMAP9 production.

### 3.6. Semi-Rational Engineering of RMAP9 for Low-Temperature Adaptation

#### 3.6.1. Selection of Candidate Residues for Mutagenesis

The trRosetta-predicted three-dimensional structure of RMAP9 is shown in Figure S6A. To evaluate the overall conformational behaviour of the simulated protein, RMAP9 was subjected to 100 ns MD simulations at 30 and 35 °C. The corresponding RMSD trajectories showed an initial conformational adjustment followed by relatively stable fluctuations during the remaining simulation period (Figure S6B), providing a basis for subsequent residue-level flexibility analysis.

Temperature-dependent local flexibility was further evaluated using residue-wise ΔRMSF, calculated as the RMSF at 35 °C minus that at 30 °C. A75, Y85 and S125 exhibited pronounced temperature-dependent fluctuations and were therefore selected as initial candidate residues (Figure S6C). Inspection of the predicted structural model further suggested that these residues are located near the predicted active-site region. The structural model was used to provide overall structural context, whereas candidate-site selection was based primarily on the residue-level dynamic response.

Rosetta-based ΔΔG scoring was subsequently used to evaluate substitutions at the three candidate positions (Table S3), and A75G, Y85G and S125A were constructed for preliminary experimental validation. Y85G exhibited apparent *K*_m_ and *V*_max_ values comparable to those of wild-type RMAP9, whereas A75G and S125A showed markedly reduced *V*_max_ values (Table S4). In addition, S125A altered the temperature-dependent activity profile within the range of 40–45 °C and affected relative activity at pH 3.0 (Figure S7).

Thus, Ser125 was not selected for site-saturation mutagenesis solely on the basis of the computational analysis. Rather, the MD-derived residue flexibility was first used to identify candidate positions, Rosetta analysis was used to evaluate possible substitutions, and preliminary experimental characterisation was then used to determine which candidate site most strongly affected the temperature-dependent biochemical behaviour of RMAP9. Because substitution at Ser125 produced a clear perturbation of the temperature–activity relationship, Ser125 was selected for subsequent site-saturation mutagenesis to determine whether alternative substitutions could shift this profile in a favourable direction.

Local conformational flexibility can facilitate catalytic conformational changes under reduced thermal energy, although excessive flexibility may also compromise the structural organisation required for efficient catalysis [40]. Therefore, the temperature-dependent flexibility identified by MD analysis was used here as a criterion for candidate-site selection rather than as direct evidence of a specific catalytic mechanism. The subsequent experimental effects of S125A provided the principal basis for prioritising Ser125 for site-saturation mutagenesis.

#### 3.6.2. Biochemical Characterisation of the Site-Saturation Variants

##### Determination of Optimum Temperature

**Figure 5:**
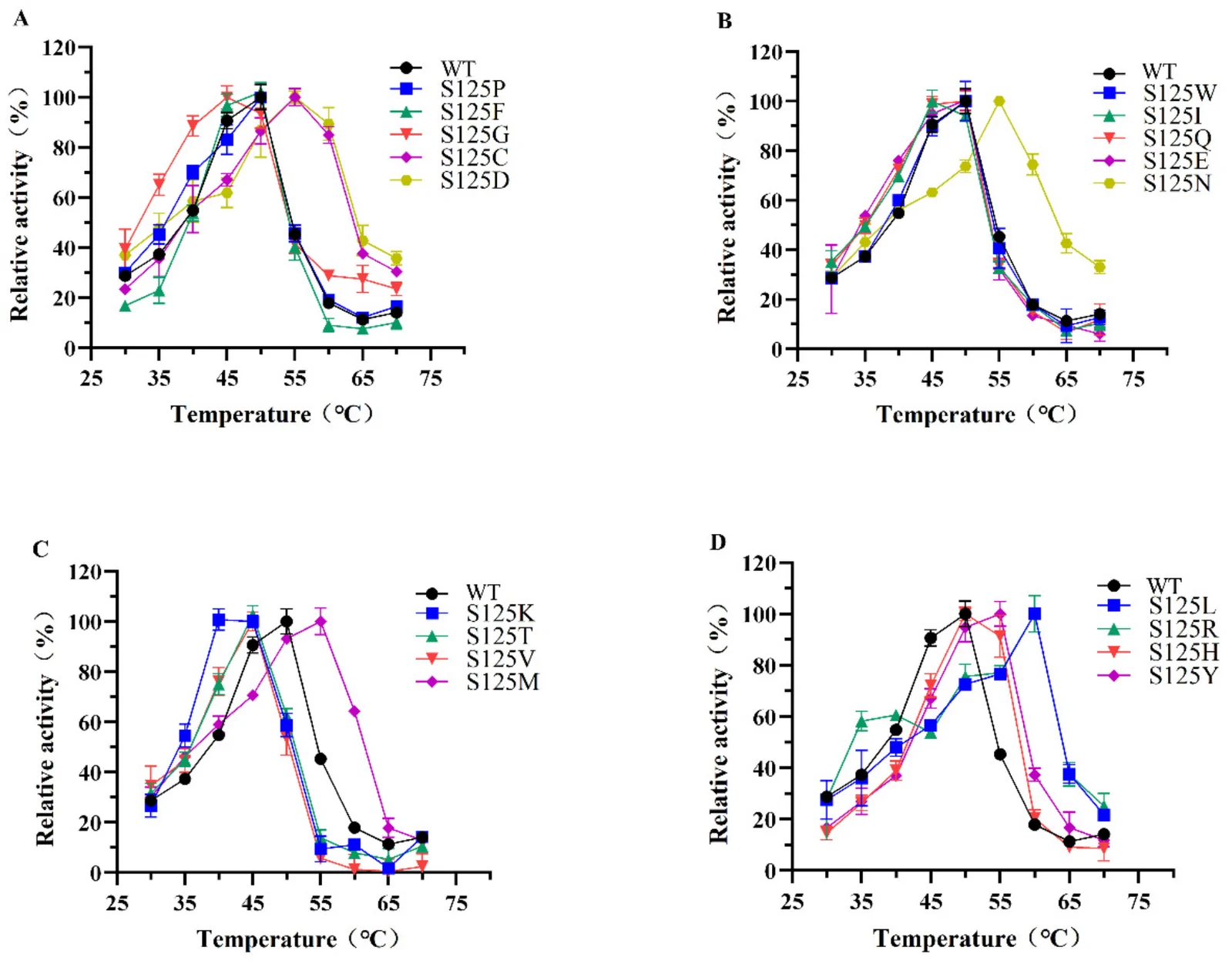
Effect of reaction temperature on the relative activities of wild-type RMAP9 and Ser125 variants: (**A**) WT, S125P, S125F, S125G, S125C and S125D; (**B**) WT, S125W, S125I, S125Q, S125E and S125N; (**C**) WT, S125K, S125T, S125V and S125M; (**D**) WT, S125L, S125R, S125H and S125Y. The maximum activity of each enzyme was defined as 100%.

Following site-saturation mutagenesis at Ser125, the optimum reaction temperatures of S125G, S125I, S125T and S125V shifted to 45 °C, whereas that of S125K shifted further to 40 °C. When the activity of each enzyme at its own optimum temperature was defined as 100%, S125G retained approximately 60–85% relative activity over the range of 35–40 °C and showed higher relative activity than wild-type RMAP9 within this temperature range. Notably, the relative enzyme activity of S125K at 40 °C was approximately 50% higher than that of the wild-type enzyme, while S125T and S125V also exhibited approximately 20% higher activities at 40 °C (Figure 5). Conversely, several other substitutions shifted the optimum reaction temperature towards higher values, with S125L exhibiting an optimum temperature of approximately 60 °C (Figure 5D). Collectively, these results demonstrate that Ser125 represents a tuneable site capable of modulating the temperature–activity profile of RMAP9 in both directions. The enhanced catalytic performance of the low-temperature-favoured variants may be associated with altered local conformational dynamics.

##### Determination of Optimum pH

**Figure 6:**
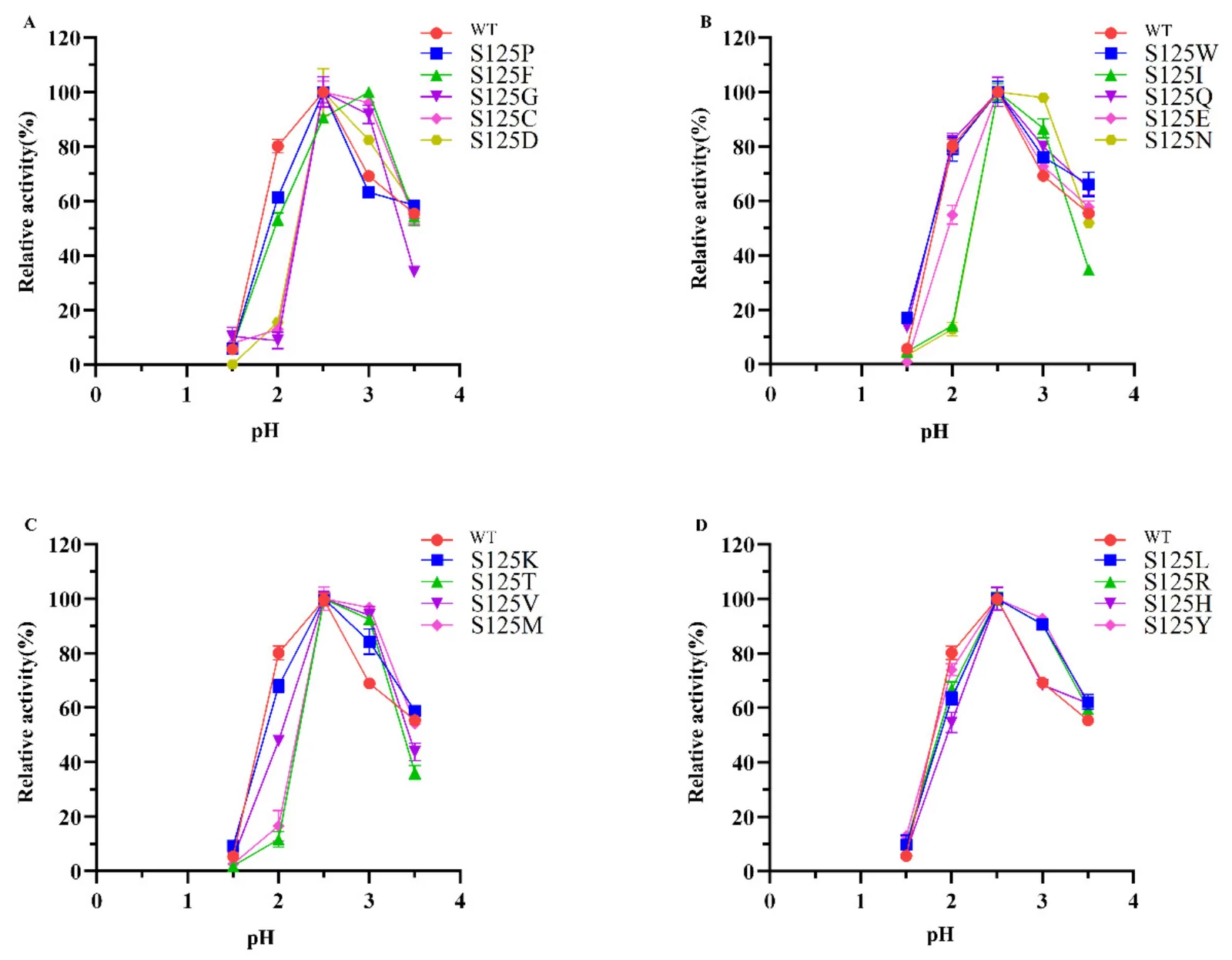
Effect of pH on the relative activities of wild-type RMAP9 and Ser125 variants: (**A**) WT, S125P, S125F, S125G, S125C and S125D; (**B**) WT, S125W, S125I, S125Q, S125E and S125N; (**C**) WT, S125K, S125T, S125V and S125M; (**D**) WT, S125L, S125R, S125H and S125Y. The maximum activity of each enzyme was defined as 100%.

With the exception of S125F, which exhibited an optimum pH of 3.0, wild-type RMAP9 and all other tested variants showed an optimum pH of 2.5, indicating that substitutions at Ser125 generally had little effect on the optimum pH of the enzyme. Most variants showed substantially reduced activity at pH 1.5, whereas several variants retained higher relative activities than wild-type RMAP9 at pH 3.0 (Figure 6). These results suggest that substitutions at Ser125 can modulate the pH-dependent activity profile of RMAP9 without substantially altering its overall acidic pH preference. As Ser125 is not one of the conserved catalytic aspartate residues, its effects on pH-dependent activity may instead arise from local structural or electrostatic changes in the vicinity of the active site; however, this proposed mechanism requires further structural validation.

##### Determination of Kinetic Parameters

The kinetic parameters of the low-temperature-favoured variants S125G, S125I, S125K, S125T and S125V were further evaluated (Table 2). S125G exhibited the highest *k*_cat_ (87.15 ± 3.75 s^−1^), exceeding that of wild-type RMAP9 (72.48 ± 3.97 s^−1^), while its *k*_cat_/*K*_m_ increased from 28.03 ± 3.99 to 33.81 ± 4.19 mL·mg^−1^·s^−1^. S125K exhibited a lower *k*_cat_ than wild-type RMAP9 but a markedly lower apparent *K*_m_, resulting in the highest *k*_cat_/*K*_m_ value among the tested enzymes (34.83 ± 8.16 mL·mg^−1^·s^−1^). S125I and S125T showed kinetic parameters relatively close to those of the wild type, whereas S125V exhibited decreases in both *k*_cat_ and *k*_cat_/*K*_m_. These results indicate that substitutions at Ser125 modulate catalytic performance through distinct kinetic effects rather than uniformly increasing catalytic turnover.

##### Stability at 30 °C

**Figure 7:**
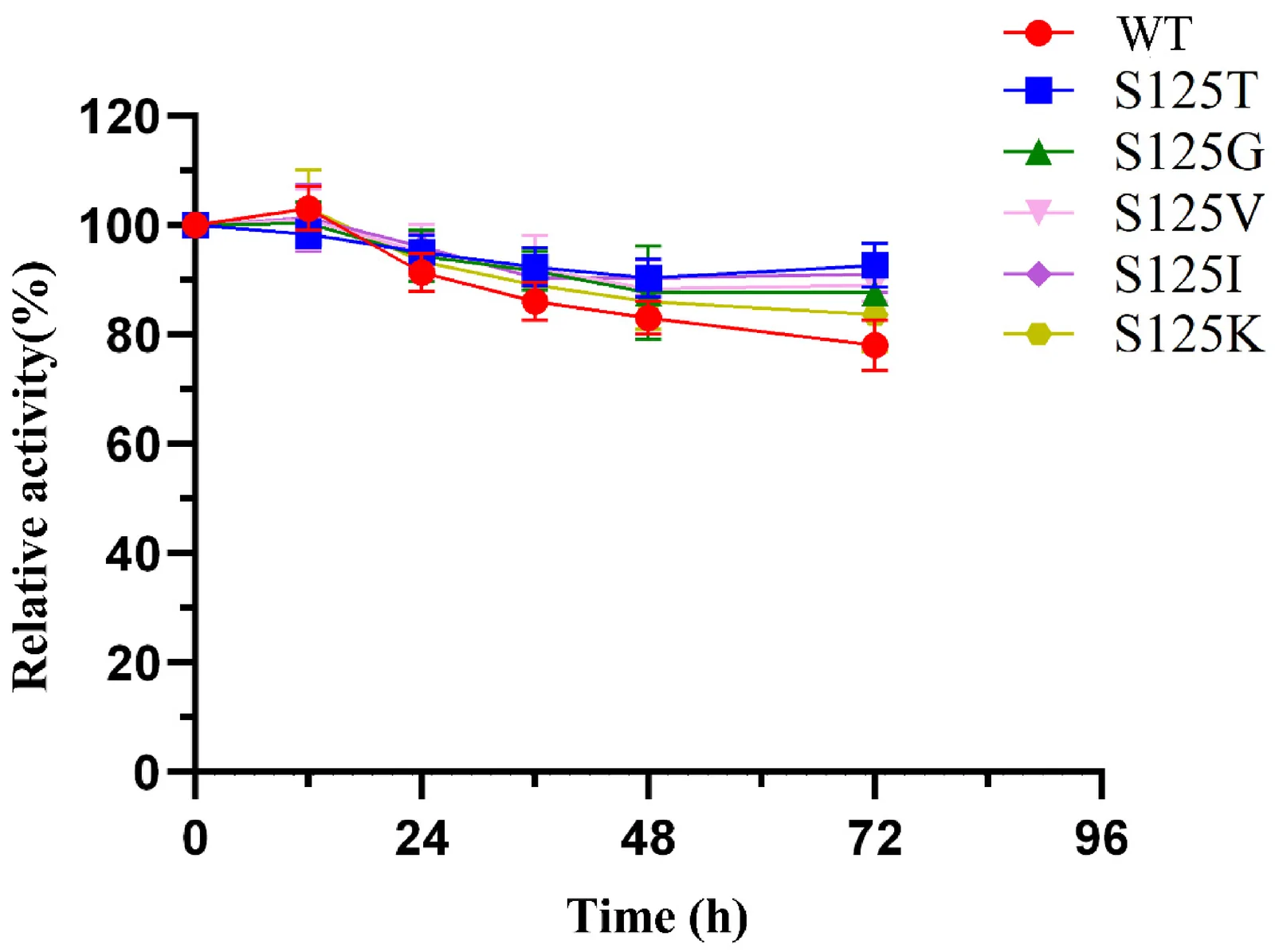
Thermal stability of wild-type RMAP9 and selected low-temperature-favoured Ser125 variants at 30 °C. Purified enzymes were incubated at 30 °C for the indicated periods, and residual activities were subsequently measured under the optimum reaction conditions of the corresponding enzymes. The activity of the corresponding enzyme preparation maintained at 4 °C was defined as 100%.

Thermal stability at 30 °C was evaluated by monitoring residual catalytic activity during prolonged incubation. After 72 h at 30 °C, all five low-temperature-favoured variants retained more than 80% of their reference activities (Figure 7), indicating that the downward shifts in optimum temperature were not accompanied by a pronounced loss of functional stability at 30 °C. Industrial ethanol fermentation with *S. cerevisiae* is generally conducted under mesophilic conditions, commonly at temperatures not exceeding approximately 37 °C, whereas further temperature elevation can adversely affect yeast growth and ethanol production [41]. Among the engineered variants, S125K shifted the optimum temperature of RMAP9 from 50 to 40 °C while maintaining substantial functional stability at 30 °C. In starch-based ethanol production, protein hydrolysis can increase the availability of free amino nitrogen for yeast and has been associated with improved fermentation performance, although the magnitude of this benefit is process-dependent [42]. Thus, the present results support the temperature-related process potential of the engineered RMAP9 variants, but their effects on nitrogen utilisation and ethanol yield require direct validation in fermentation systems.

## 4. Conclusions

In this study, an acid protease-producing *Rhodotorula mucilaginosa* strain, TSM-6, was isolated from mangrove sediment, and the acid protease RMAP9 was identified. Both methanol-inducible and constitutive recombinant production systems were established in *K. phaffii*. Through EXG1-derived pre-region engineering, tandem expression-cassette assembly and optimisation of shake-flask cultivation conditions, extracellular acid protease activity increased from 96.52 ± 2.53 U/mL for the α-factor-based constitutive reference to 312.07 ± 18.37 U/mL, corresponding to an overall increase of approximately 223%. Molecular dynamics simulations combined with experimental mutagenesis further demonstrated that Ser125 can modulate the temperature–activity profile of RMAP9 in both directions. Among the variants with lower optimum temperatures, S125K shifted the optimum temperature from 50 to 40 °C and retained substantially higher relative activity than wild-type RMAP9 at 40 °C, while the selected variants retained more than 80% residual activity after 72 h at 30 °C. Overall, this study provides an enhanced methanol-free production strategy for RMAP9 and identifies Ser125 as a useful target for temperature-adaptation engineering. These findings support further process development and evaluation of RMAP9 under practical ethanol-fermentation conditions.

## Figures and Tables

**Table 1:** L_9_(3^4^) orthogonal design and extracellular acid protease activity.

No.	A (Glucose)	B (Beef Extract)	C (Yeast Extract)	Enzyme Activity (U/mL)
1	1	1	1	98.01
2	1	2	2	121.01
3	1	3	3	92.99
4	2	1	2	120.23
5	2	2	3	110.12
6	2	3	1	91.55
7	3	1	3	135.29
8	3	2	1	117.61
9	3	3	2	82.65
K1	104.003	117.843	102.390	
K2	107.300	116.247	107.963	
K3	111.850	89.063	112.800	
Range R	7.847	28.780	10.410	

K1–K3 represent the mean enzyme activities at levels 1–3, respectively; R represents the range.

**Table 2:** Apparent kinetic parameters of wild-type RMAP9 and selected Ser125 variants.

Enzyme	*K*_m_ (mg·mL^−1^)	*V_m__a__x_* (μmol·min^−1^·mg^−1^)	*k_cat_* (s^−1^)	*k_cat_*/*K_m_* (mL·mg^−1^·s^−1^)
RMAP9	2.58 ± 0.34	127.90 ± 7.00	72.48 ± 3.97	28.03 ± 3.99
S125G	2.57 ± 0.30	153.80 ± 6.62	87.15 ± 3.75	33.81 ± 4.19
S125I	2.34 ± 0.31	124.30 ± 6.55	70.44 ± 3.71	30.00 ± 4.26
S125K	1.50 ± 0.33	92.38 ± 7.56	52.35 ± 4.28	34.83 ± 8.16
S125T	2.58 ± 0.31	135.40 ± 6.85	76.73 ± 3.88	29.72 ± 3.87
S125V	1.54 ± 0.38	60.17 ± 5.29	34.10 ± 3.00	22.01 ± 5.74

## Data Availability

The original contributions presented in this study are included in the article and Supplementary Materials. Further inquiries can be directed to the corresponding author.

## Supplementary Materials

The following supporting information can be downloaded at: https://www.mdpi.com/article/10.3390/biotech15040087/s1, Figure S1: Colony and cell morphological characteristics of strain TSM-6; Table S1: Carbon assimilation profile of strain TSM-6; Figure S2: Agarose gel electrophoresis of PCR products used for molecular identification of strain TSM-6; Figure S3: Neighbour-joining phylogenetic tree of strain TSM-6 based on the D1/D2 domain of the 26S rRNA gene; Table S2: Predicted sequence and physicochemical characteristics of the putative acid proteases identified in *R. mucilaginosa* TSM-6; Figure S4: Multiple sequence alignment of the putative acid proteases identified in *R. mucilaginosa* TSM-6; Figure S5: Verification and heterologous expression analysis of candidate acid proteases in *K. phaffii* GS115; Figure S6: Structural model and molecular dynamics analysis of RMAP9; Table S3: Rosetta ΔΔG scores for substitutions at A75, Y85 and S125 of RMAP9; Figure S7: Biochemical properties of wild-type RMAP9 and the preliminary variants A75G, Y85G and S125A; Table S4: Apparent kinetic parameters of wild-type RMAP9 and the preliminary variants A75G, Y85G and S125A; Table S5: Primers used in this study.

## References

[1] Theron L.W., Divol B. (2014). Microbial aspartic proteases: Current and potential applications in industry. Appl. Microbiol. Biotechnol..

[2] Dos Santos Aguilar J.G., Sato H.H. (2018). Microbial proteases: Production and application in obtaining protein hydrolysates. Food Res. Int..

[3] Solanki P., Putatunda C., Kumar A., Bhatia R., Walia A. (2021). Microbial proteases: Ubiquitous enzymes with innumerable uses. 3 Biotech.

[4] Zhao X., Du G., Zou H., Fu J., Zhou J., Chen J. (2013). Progress in preventing the accumulation of ethyl carbamate in alcoholic beverages. Trends Food Sci. Technol..

[5] Tien T.N., Nguyen T.C., Nguyen C.N., Nguyen T.T., Pham T.A., Pham N.H., Chu-Ky S. (2022). Protease increases ethanol yield and decreases fermentation time in no-cook process during very-high-gravity ethanol production from rice. Process Biochem..

[6] Mandujano-González V., Villa-Tanaca L., Anducho-Reyes M.A., Mercado-Flores Y. (2016). Secreted fungal aspartic proteases: A review. Rev. Iberoam. Micol..

[7] Juturu V., Wu J.C. (2018). Heterologous protein expression in *Pichia pastoris*: Latest research progress and applications. ChemBioChem.

[8] Chi Z., Chi Z., Zhang T., Liu G., Li J., Wang X. (2009). Production, characterization and gene cloning of the extracellular enzymes from the marine-derived yeasts and their potential applications. Biotechnol. Adv..

[9] Lario L.D., Chaud L., Almeida M.G., Converti A., Sette L.D., Pessoa A. (2015). Production, purification, and characterization of an extracellular acid protease from the marine Antarctic yeast *Rhodotorula mucilaginosa* L7. Fungal Biol..

[10] Chaud L.C.S., Lario L.D., Bonugli-Santos R.C., Sette L.D., Pessoa Junior A., Felipe M.G.A. (2016). Improvement in extracellular protease production by the marine Antarctic yeast *Rhodotorula mucilaginosa* L7. New Biotechnol..

[11] Nimsi K.A., Manjusha K., Hatha A.A.M., Kathiresan K. (2023). Diversity, distribution, and bioprospecting potentials of manglicolous yeasts: A review. FEMS Microbiol. Ecol..

[12] Lv W., Cai M. (2025). Advancing recombinant protein expression in *Komagataella phaffii*: Opportunities and challenges. FEMS Yeast Res..

[13] Vogl T., Glieder A. (2013). Regulation of *Pichia pastoris* promoters and its consequences for protein production. New Biotechnol..

[14] Puxbaum V., Mattanovich D., Gasser B. (2015). Quo vadis? The challenges of recombinant protein folding and secretion in *Pichia pastoris*. Appl. Microbiol. Biotechnol..

[15] Liang S., Li C., Ye Y., Lin Y. (2013). Endogenous signal peptides efficiently mediate the secretion of recombinant proteins in *Pichia pastoris*. Biotechnol. Lett..

[16] Aw R., Polizzi K.M. (2013). Can too many copies spoil the broth?. Microb. Cell Fact..

[17] Looser V., Bruhlmann B., Bumbak F., Stenger C., Costa M., Camattari A., Fotiadis D., Kovar K. (2015). Cultivation strategies to enhance productivity of *Pichia pastoris*: A review. Biotechnol. Adv..

[18] Yegin S., Fernandez-Lahore M., Gama Salgado A.J., Guvenc U., Goksungur Y., Tari C. (2011). Aspartic proteinases from *Mucor* spp. in cheese manufacturing. Appl. Microbiol. Biotechnol..

[19] Cripwell R.A., Favaro L., Viljoen-Bloom M., van Zyl W.H. (2020). Consolidated bioprocessing of raw starch to ethanol by *Saccharomyces cerevisiae*: Achievements and challenges. Biotechnol. Adv..

[20] Siddiqui K.S. (2015). Some like it hot, some like it cold: Temperature dependent biotechnological applications and improvements in extremophilic enzymes. Biotechnol. Adv..

[21] Herman R.A., Ayepa E., Zhang W.-X., Li Z.-N., Zhu X., Ackah M., Yuan S.-S., You S., Wang J. (2024). Molecular modification and biotechnological applications of microbial aspartic proteases. Crit. Rev. Biotechnol..

[22] Wijma H.J., Janssen D.B. (2013). Computational design gains momentum in enzyme catalysis engineering. FEBS J..

[23] Lutz S. (2010). Beyond directed evolution—Semi-rational protein engineering and design. Curr. Opin. Biotechnol..

[24] Bornscheuer U.T., Huisman G.W., Kazlauskas R.J., Lutz S., Moore J.C., Robins K. (2012). Engineering the third wave of biocatalysis. Nature.

[25] Yang J., Anishchenko I., Park H., Peng Z., Ovchinnikov S., Baker D. (2020). Improved protein structure prediction using predicted interresidue orientations. Proc. Natl. Acad. Sci. USA.

[26] Abraham M.J., Murtola T., Schulz R., Páll S., Smith J.C., Hess B., Lindahl E. (2015). GROMACS: High performance molecular simulations through multi-level parallelism from laptops to supercomputers. SoftwareX.

[27] Huang J., Rauscher S., Nawrocki G., Ran T., Feig M., de Groot B.L., Grubmüller H., MacKerell A.D. (2017). CHARMM36m: An improved force field for folded and intrinsically disordered proteins. Nat. Methods.

[28] Leaver-Fay A., Tyka M., Lewis S.M., Lange O.F., Thompson J., Jacak R., Kaufman K., Renfrew P.D., Smith C.A., Sheffler W. (2011). Rosetta3: An object-oriented software suite for the simulation and design of macromolecules. Methods Enzymol..

[29] Kellogg E.H., Leaver-Fay A., Baker D. (2011). Role of conformational sampling in computing mutation-induced changes in protein structure and stability. Proteins.

[30] Takagi S., Tsutsumi N., Terui Y., Kong X., Yurimoto H., Sakai Y. (2019). Engineering the expression system for *Komagataella phaffii* (*Pichia pastoris*): An attempt to develop a methanol-free expression system. FEMS Yeast Res..

[31] Zou C., Lu L., Wang S., Zhang C., Chen X., Lin Y., Huang Y. (2022). The α-mating factor secretion signals and endogenous signal peptides for recombinant protein secretion in *Komagataella phaffii*. Biotechnol. Biofuels Bioprod..

[32] Karaoğlan M. (2023). Alternative secretory signal sequences for recombinant protein production in *Pichia pastoris*. Enzym. Microb. Technol..

[33] Ito Y., Ishigami M., Hashiba N., Nakamura Y., Terai G., Hasunuma T., Ishii J., Kondo A. (2022). Avoiding entry into intracellular protein degradation pathways by signal mutations increases protein secretion in *Pichia pastoris*. Microb. Biotechnol..

[34] Zhang Y.-S., Gong J.-S., Jiang J.-Y., Xu Z.-H., Shi J.-S. (2024). Engineering protein translocation and unfolded protein response enhanced human PH-20 secretion in *Pichia pastoris*. Appl. Microbiol. Biotechnol..

[35] Zhu T., Guo M., Tang Z., Zhang M., Zhuang Y., Chu J., Zhang S. (2009). Efficient generation of multi-copy strains for optimizing secretory expression of porcine insulin precursor in yeast *Pichia pastoris*. J. Appl. Microbiol..

[36] Hohenblum H., Gasser B., Maurer M., Borth N., Mattanovich D. (2004). Effects of gene dosage, promoters, and substrates on unfolded protein stress of recombinant *Pichia pastoris*. Biotechnol. Bioeng..

[37] Schwarzhans J.-P., Wibberg D., Winkler A., Luttermann T., Kalinowski J., Friehs K. (2016). Integration event induced changes in recombinant protein productivity in *Pichia pastoris* discovered by whole genome sequencing and derived vector optimization. Microb. Cell Fact..

[38] La Barbera Kastberg L., Jacobsen I.H., Özdemir E., Workman C.T., Jensen M.K., Förster J. (2025). Characterizing heterologous protein burden in *Komagataella phaffii*. FEMS Yeast Res..

[39] Gündüz Ergün B., Berrios J., Binay B., Fickers P. (2021). Recombinant protein production in *Pichia pastoris*: From transcriptionally redesigned strains to bioprocess optimization and metabolic modelling. FEMS Yeast Res..

[40] Collins T., Feller G. (2023). Psychrophilic enzymes: Strategies for cold-adaptation. Essays Biochem..

[41] Robak K., Balcerek M. (2020). Current state-of-the-art in ethanol production from lignocellulosic feedstocks. Microbiol. Res..

[42] Li J., Zhao R., Xu Y., Wu X., Bean S.R., Wang D. (2022). Fuel ethanol production from starchy grain and other crops: An overview on feedstocks, affecting factors, and technical advances. Renew. Energy.

