# Supplementary material for: High-Level Constitutive Secretory Expression and Low-Temperature Adaptation Engineering of the Acid Protease RMAP9 from the Mangrove-Derived Yeast Rhodotorula mucilaginosa
Source: BioTech (Basel). 2026 Oct 6;15(4):87. doi: 10.3390/biotech15040087 (PMC13702498; doi:10.3390/biotech15040087)
Supplement: Supplementary file 1 [file biotech-15-00087-s001.zip › biotech-4547363-supplementary.pdf]

Supplementary Materials: High-Level Constitutive Secretory Expression and Low-Temperature Adaptation Engineering of the Acid Protease RMAP9 from the Mangrove-Derived Yeast *Rhodotorula mucilaginosa*

Yibo Zhang, Jiacheng Cao, Mingming Luo, Zhijie Huang, Yu Tang, Zhizheng Zhao, Jian Lu

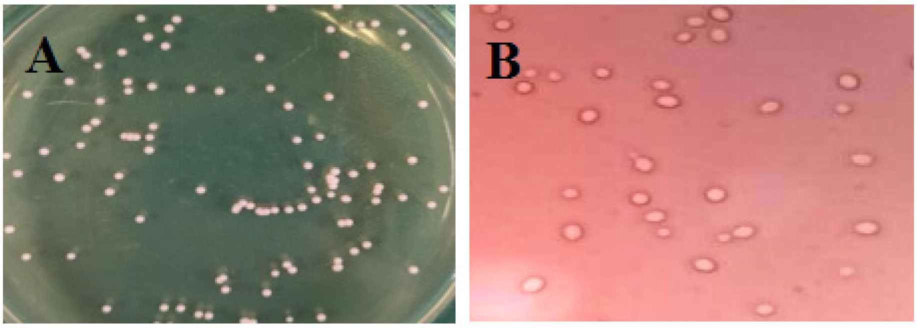

Figure S1. Colony and cell morphological characteristics of strain TSM-6: (A) colony morphology after cultivation on YPD agar; (B) cell morphology of strain TSM-6 observed by light microscopy.

Table S1. Carbon assimilation profile of strain TSM-6.

Carbon sources	Results	Carbon sources	Results
Glucose	+	Soluble starch	+
Sucrose	+	Raffinose	-
Cellobiose	-	α-lactose	+
Maize xylan	-	Galactose	+
Maltose	-		

Note: +: positive; -: negative.

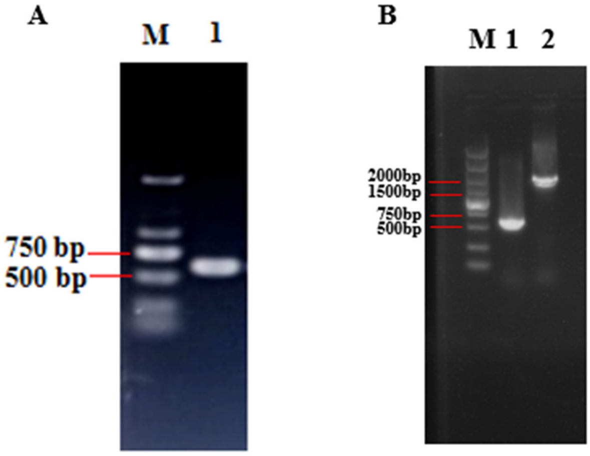

Figure S2. Agarose gel electrophoresis of PCR products used for molecular identification of strain TSM-6: (A) M: DL2000 DNA Marker; 1: PCR product of strain TSM-6 D1/D2 domain of the 26S rRNA gene; (B) M: DL5000 DNA Marker; 1: PCR product of the ITS region of strain TSM-6; 2: PCR product of the 18S rRNA gene of strain TSM-6.

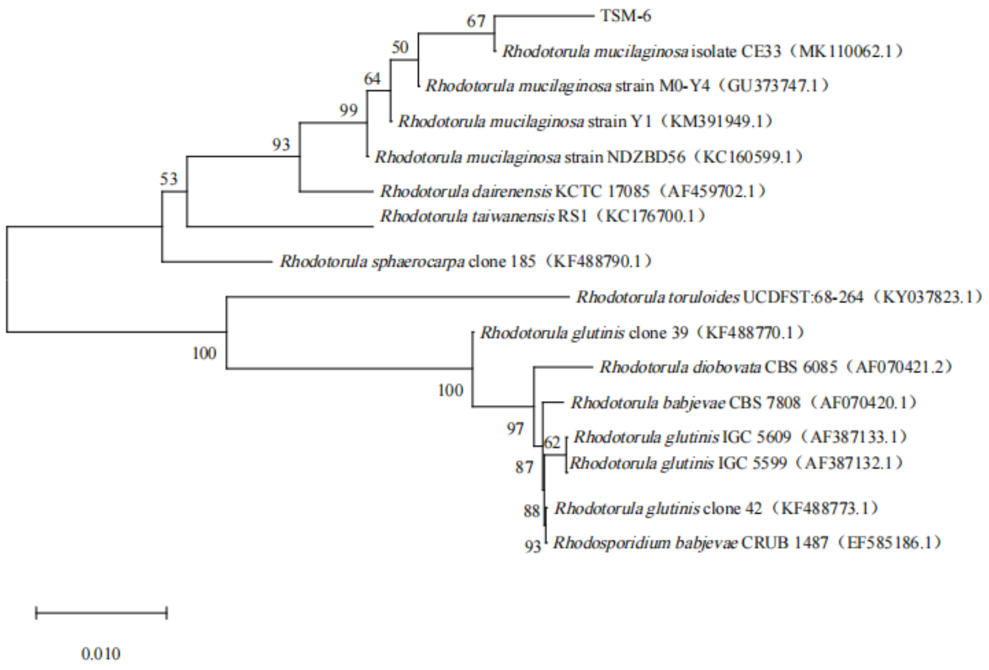

Figure S3. Neighbour-joining phylogenetic tree of strain TSM-6 based on the D1/D2 domain of the 26S rRNA gene.

Table S2. Predicted sequence and physicochemical characteristics of the putative acid proteases identified in *R. mucilaginosa* TSM-6.

Gene	CDS length(bp)	Protein length (aa)	Predicted signal peptide (aa)	Mature-protein length (aa)	Predicted mature-protein MW (kDa)	Mature-protein theoretical pI
rmap1	2127	708	1–39	456	50	4.53
rmap2	1956	651	1–24	512	56	3.96
rmap3	1329	442	1–28	304	33	4.16
rmap4	1404	467	1–21	334	36	5.31
rmap5	1443	480	1–25	369	40	4.02
rmap6	1308	435	1–25	328	36	5.31
rmap7	1602	533	1–20	331	36	4.81
rmap8	1326	441	1–28	307	33	4.17
rmap9	1278	425	1–20	316	34	5.69
rmap10	1740	579	1–20	460	50	4.49

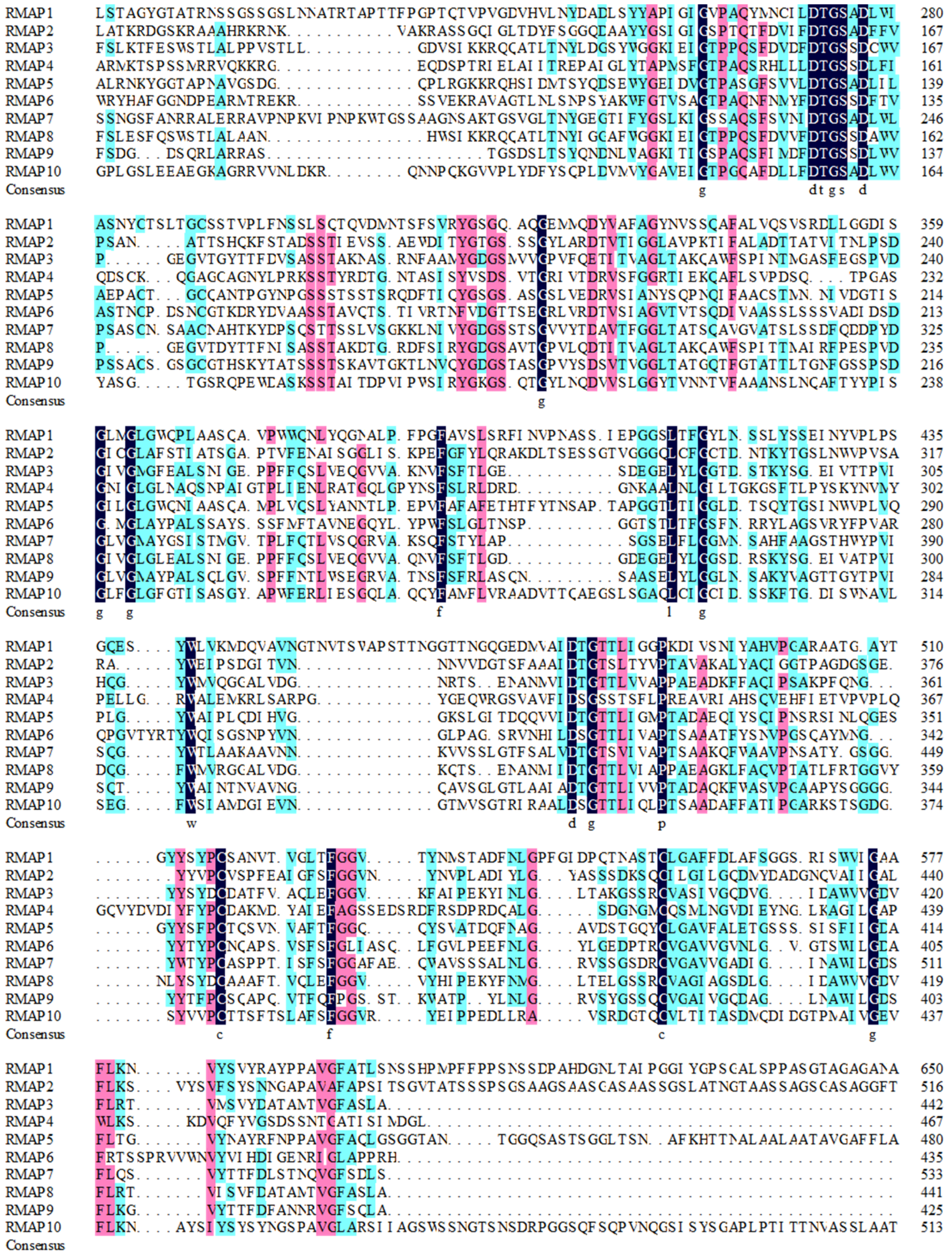

Figure S4. Multiple sequence alignment of the putative acid proteases identified in *R. mucilaginosa* TSM-6.

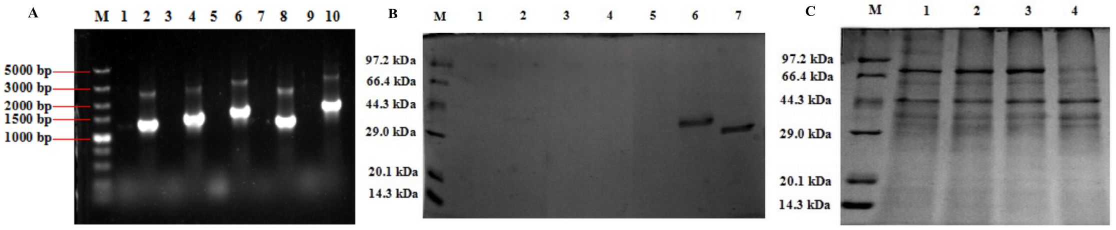

Figure S5. Verification and heterologous expression analysis of candidate acid proteases in *K. phaffii* GS115. (A) Genomic PCR verification of the five methanol-inducible recombinant strains. M, DL5000

DNA marker; lanes 1, 3, 5, 7 and 9, PCR products of the GS115/pPIC9K vector controls; lanes 2, 4, 6, 8 and 10, PCR products of GS115/pPIC9K-*rmap3*, GS115/pPIC9K-*rmap5*, GS115/pPIC9K-*rmap7*, GS115/pPIC9K-*rmap9* and GS115/pPIC9K-*rmap10*, respectively. The corresponding target products were approximately 1.3, 1.4, 1.6, 1.2 and 1.7 kb, respectively. (B) SDS-PAGE analysis of culture supernatants from the methanol-inducible recombinant strains. M, Premixed Protein Marker (Low); lanes 1 and 5, GS115/pPIC9K vector controls; lane 2, RMAP3; lane 3, RMAP5; lane 4, RMAP10; lane 6, RMAP7; lane 7, RMAP9. Distinct bands corresponding to RMAP7 and RMAP9 were detected at approximately 36 and 34 kDa, respectively. (C) SDS-PAGE analysis of cell lysates from strains lacking obvious extracellular target bands. M, Premixed Protein Marker (Low); lane 1, GS115/pPIC9K vector control; lane 2, RMAP3; lane 3, RMAP5; lane 4, RMAP10. No obvious target bands corresponding to RMAP3, RMAP5 or RMAP10 were detected in the cell lysates.

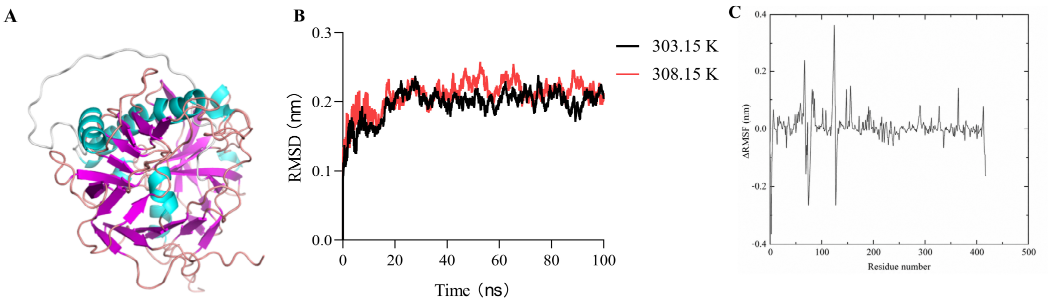

Figure S6. Structural model and molecular dynamics analysis of RMAP9. (A) Three-dimensional structure of RMAP9 predicted using trRosetta. (B) RMSD profiles of RMAP9 during 100 ns molecular dynamics simulations at 30 and 35 °C (303.15 and 308.15 K, respectively). (C) Residue-wise ΔRMSF profile calculated as the RMSF at 35 °C minus the RMSF at 30 °C. A75, Y85 and S125 were selected as candidate residues based on their pronounced temperature-dependent fluctuations observed in the ΔRMSF analysis.

Table S3. Rosetta ΔΔG scores for substitutions at A75, Y85 and S125 of RMAP9.

Substituting residue	A75	Y85	S125
Gly	-10.47	4.28	3.89
Ala	0	0.89	7.79
Val	1.68	2.03	0.87
Leu	-2.14	-0.59	-0.69
Ile	4	-1.52	2.21
Phe	0.93	0.94	0.36
Trp	4.77	0.78	4.73
Tyr	-0.78	0	2.21
Asp	2.56	1.54	0.25
Asn	4.13	0.79	-0.86
Glu	2.75	2.18	1.21
Lys	0.53	-1.41	3.25
Gln	1.75	0.63	-3.18
Met	2.61	-1.38	1.02
Ser	-1.87	0.64	0
Thr	0.96	1.34	4.61
Cys	3.78	0.73	3.12
Pro	-2.19	1.40	-0.54
His	2.95	-0.97	0.98
Arg	3.21	-0.84	2.36

*Values are reported as Rosetta ΔΔG scores without assignment of a physical energy unit.

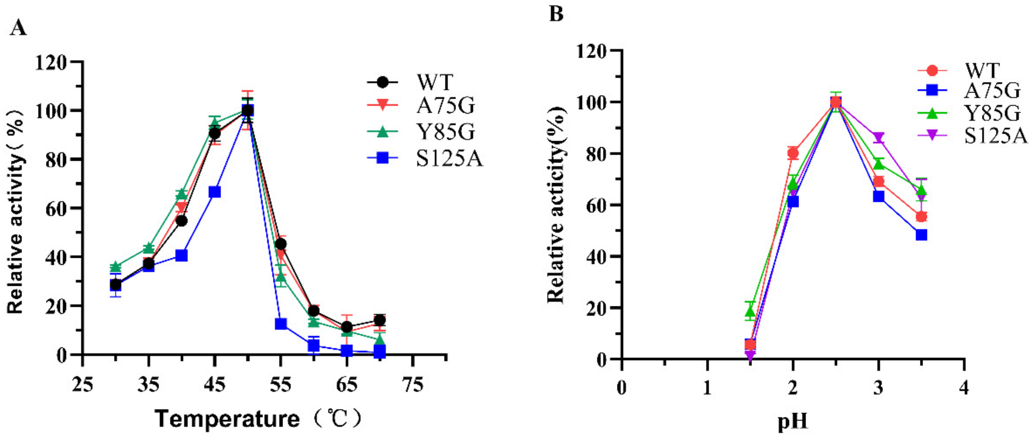

Figure S7. Biochemical properties of wild-type RMAP9 and the preliminary variants A75G, Y85G and S125A: (A) effect of reaction temperature on relative activity; (B) effect of pH on relative activity. The maximum activity of each enzyme was defined as 100%.

Table S4. Apparent kinetic parameters of wild-type RMAP9 and the preliminary variants A75G, Y85G and S125A.

Enzyme	K_m (mg·mL^{-1})	V_{max} (*μmol·min^{-1}·mg^{-1}*)
RMAP9	2.586±0.34	127.90±7.00
A75G	2.422±0.33	72.21±4.01
Y85G	2.720±0.32	130.00±6.60
S125A	2.139±0.29	62.10±3.24

Table S5. Primers used in this study.

Primer	Sequence (5'-3')
rmap1-F	ATGGCACCCTCTCGAGC
rmap1-R	TCACGTCAACAACCCGAG
rmap2-F	ATGCTCTTCTCGTCCGC
rmap2-R	CTACAGGATCAATCCGAGAC
rmap3-F	ATGATCGCTGCGGGTTC
rmap3-R	TCAAGCGAGGGAGGCGAA
rmap4-F	ATGGTCCAATTTTCTATCCT
rmap4-R	CTACAACCCATCCATGAT
rmap5-F	ATGCCCACAACATCTGCC
rmap5-R	TTATGCGAGGAAGAATGCAC
rmap6-F	ATGCTCGCGTCTTCTGCCCT
rmap6-R	TCAGTGCCGGGGCGGAG
rmap7-F	ATGCGTACCGCCGCCCTC
rmap7-R	TTACGAGAGGTCGGAGAAGCCAAC
rmap8-F	ATGTTCGCTTCGGGTTCTGT
rmap8-R	TCACGCGAGGGAAGCGAAGC
rmap9-F	ATGCCTTCGCTCGTCGCC
rmap9-R	CTAAGCGAGCTGAGAGAAGCC
rmap10-F	ATGTTGCCGTTCCTGACGG
rmap10-R	TCAGAGGAGTGCCGCGC
RT *gap*-F	GGTATTAACGGTTTCGGACGTATTG
RT *gap*-R	GATGTTGACAGGGTCTCTCTCTTGG
RT*rmap9*-F	CCAGAACGACAACCTGTGGGCGGGC
RT*rmap9*-R	GTAGACCGGTCCAGAAGCGGTCGAG

18S-F	CCAACCTGGTTGATCCTGCCAG
18S-R	CCTTGTTACGACTTCACCTTCCTCT
ITS-F	TCCGTAGGTGAACCTGCGG
ITS-R	TCCTCCGCTTATTGATATGC
26S-F	GCATATCAATAAGCGGAGGAAAAG
26S-R	GGTCCGTGTTTCAAGACGG
DSE4-F	GAACAACTATTTCGAAACGATGTCATTCTCTTCCAACGTG
DSE4-R	TAGTGTTGACTGGAGCAGCTCCACTGACTATATTGGTCAACA
EXG1-F	TGAACAACTATTTCGAAACGATGGGGATGAACTTGTACCT
EXG1-R	GTAGTGTTGACTGGAGCAGCACTGCATAGGCTGGC
FLO10-F	TGAACAACTATTTCGAAACGATGTTTGAGAAGAGTAAATTTGTGG
FLO10-R	TAGTGTTGACTGGAGCAGCACCAAGGACACAAAATAGCT
PHO5-F	AACAACTATTTCGAAACGATGTTTTCTCCTATTCTAAGTCTG
PHO5-R	TAGTGTTGACTGGAGCAGCAAAGACTGATTGGAGAGTAGC
A75G-F	TCGTCGAGGGTCGACCGGTTCCGACAG
A75G-R	CGGTCGACCCTCGACGAGCAAGTCGCT
Y85G-F	CACGTCTGGCCAGAACGACAACCTGTGGGC
Y85G-R	TTCTGGCCAGACGTGAGACTGTCGGAACC
S125A-F	CTCGGGCGCTGGCTGCGGCACGCACTCCAA
S125A-R	CAGCCAGCCGCCCGAGCAAGCACTCGAAGGG
S125L-F	GAGTGCTTGCTCGGGCTTAGGCTGCGGCACGCACTCC
S125I-F	GAGTGCTTGCTCGGGCATTGGCTGCGGCACGCACTCC
S125M-F	GAGTGCTTGCTCGGGCATGGGCTGCGGCACGCACTCC
S125V-F	GAGTGCTTGCTCGGGCGTTGGCTGCGGCACGCACTCC
S125T-F	GAGTGCTTGCTCGGGCACTGGCTGCGGCACGCACTCC
S125Y-F	GAGTGCTTGCTCGGGCTATGGCTGCGGCACGCACTCC
S125H-F	GAGTGCTTGCTCGGGCCATGGCTGCGGCACGCACTCC
S125Q-F	GAGTGCTTGCTCGGGCCAAGGCTGCGGCACGCACTCC
S125N-F	GAGTGCTTGCTCGGGCAATGGCTGCGGCACGCACTCC
S125K-F	GAGTGCTTGCTCGGGCAAAGGCTGCGGCACGCACTCC
S125D-F	GAGTGCTTGCTCGGGCGATGGCTGCGGCACGCACTCC
S125E-F	GAGTGCTTGCTCGGGCGAAGGCTGCGGCACGCACTCC
S125W-F	GAGTGCTTGCTCGGGCTGGGGCTGCGGCACGCACTCC
S125R-F	GAGTGCTTGCTCGGGCCGTGGCTGCGGCACGCACTCC
S125G-F	GAGTGCTTGCTCGGGCGGTGGCTGCGGCACGCACTCC
S125C-F	GAGTGCTTGCTCGGGCTGTGGCTGCGGCACGCACTCC
S125P-F	GAGTGCTTGCTCGGGCCCTGGCTGCGGCACGCACTCC
S125F-F	GAGTGCTTGCTCGGGCTTTGGCTGCGGCACGCACTCC
S125-R	CCGAGCAAGCACTCGAAGGG
